# Biomimetic-Functionalized, Tannic Acid-Templated Mesoporous Silica as a New Support for Immobilization of NHase

**DOI:** 10.3390/molecules22101597

**Published:** 2017-09-25

**Authors:** Jun-kai Gao, Zi-jun Zhang, Yan-jun Jiang, Yan Chen, Shu-feng Gao

**Affiliations:** 1 School of Port and Transportation Engineering, Zhejiang Ocean University, Zhoushan 316022, China; gaojk@zjou.edu.cn (J.-k.G.); 18857088135@163.com (Z.-j.Z.); 2 School of Chemical Engineering and Technology, Hebei University of Technology, Tianjin 300130, China; yanjunjiang@hebut.edu.cn; 3 YinzhouKefeng New Material of Polymer Co. Ltd., Ningbo 315100, China; gsf324@163.com

**Keywords:** dopamine, mesoporous silica, tannic acid, immobilization, nitrile hydratase

## Abstract

Tannic acid-templated mesoporous silica (TAMS) was synthesized using a simple nonsurfactant template method and dopamine-functionalized TAMS (Dop-TAMS), which was prepared via a biomimetic coating, was developed as a new support for immobilization of NHase (NHase@Dop-TAMS). The Dop-TAMS was thoroughly characterized by the transmission electron microscopy (TEM), scanning electron microscopy (SEM), Brunauer–Emmett–Teller (BET), and Fourier transform infrared (FT-IR) and the results showed that the Dop-TAMS possessed sufficiently large pore size and volume for the accommodation of NHase. Studying the thermal stability, storage, shaking stability, and pH stability of the free and immobilized NHase indicated that the catalytic properties of NHase@Dop-TAMS were significantly enhanced. Moreover, the NHase@Dop-TAMS exhibited good reusability. All the results demonstrated that Dop-TAMS could be used as an excellent matrix for the immobilization of NHase.

## 1. Introduction

Enzymes have a wide range of applications, including in disease diagnosis, clinical treatment, and in the food industry, due to several advantages, such as mild reaction conditions, high efficiency, and specificity [1,2]. However, the application of free enzymes is still limited by low environmental adaptability, poor operational stability, and poor recyclability [3]. It is important, therefore, to overcome these drawbacks and the immobilization of enzymes on solid carriers has been demonstrated to improve their adaptability to changes in surroundings and raise their recycling rate [4,5]. The methods proposed for enzyme immobilization are distinguished as either physical or chemical. Physical methods involve physisorption and entrapment and chemical methods are divided into ion binding, covalent binding, and cross-linking [6,7].

In the preparation of immobilized enzymes, it is essential to select an appropriate carrier to improve the operational stability of the biocatalyst [8,9]. Many carriers of enzymes have been studied, including polymer supports, metallic and glass materials, organic and inorganic nanometer compound materials, carbon nanotubes, and colloidal supports [10]. Among the numerous carriers, mesoporous silica has attracted significant research attention due to its large specific surface area, adjustable pore structure with ordered arrangement, good mechanical stability, and biocompatibility [11,12]. For example, Hung et al. utilized 3-aminopropyltrimethoxysilane and glutaraldehyde-functionalized mesoporous silica nanoparticles (IBN-4) as the carrier of Horseradish peroxidase (HRP) and the results exhibited that IBN-4 greatly increased stability and activity of HRP enzymes [13]. Ali et al. covalently immobilized the *Candida rugosa* lipase on fibrous silica nanoparticles (KCC-1), and the resulting immobilized lipase exhibited wider reaction temperatures and pH regionsin comparison to the free lipase [14]. Gao et al. synthesized monodisperse core-shell magnetic organosilicanano flowers as the support for *Candida antarctica* lipase B (CALB) immobilization and the study results showed that the the resulting immobilized enzyme (CALB/nanoflowers) exhibited improved pH stability, improved thermal stability, and satisfactory storage stability [15]. Li et al. Developed hierarchically macro/mesoporous silica sphere with structural macropores and skeleton mesopores for immobilization of catalase. The study results indicated that the immobilized catalase showed comparable catalytic efficiency to the free catalase and possessed appreciable reusability [16]. However, the traditional synthetic method for mesoporous silica involves the application of surfactant, such as triblock copolymers and cetyltrimethylammonium bromide [17,18,19] and these surfactant templating agents are toxic, expensive, and difficult to remove [20,21]. Therefore, a templating agent that is cheap, non-toxic, and easy to remove is needed. Recent research has found that tannic acid may be a suitable template agent. Tannic acid is a class of polyphenol compound with complex structure and is found in numerous plants [22,23]. It is not only cheap and environmental friendly, but can also be dissolved in water or alcohol. Therefore, when tannic acid is used as template agent, it can be easily removed by water or alcohol. Additionally, mesoporous silica synthesized with tannic acid as template agent had large interconnected mesopores, which benefited the accommodation of enzymes [18]. Considering these advantages, tannic acid-templated mesoporous silica (TAMS) has great potential for the immobilization of enzymes in practical applications.

Typical methods for immobilizing enzymes in mesoporous silicas rely on grafting suitable functional groups on their surfaces, which can react with the enzymes by covalent conjugation [24,25]. However, such immobilization strategies are usually accompanied by enzyme deactivation [25]. The utilization of dopamine as a surface functionalization reagent has attracted much attention because it is facile, simple, and cost-effective and the dopamine molecules contain both primary amine and catechol, which have the ability to immobilize biomolecules [26,27]. Furthermore, it avoids calcination of the TAMS during synthesis and therefore allows more silanol groups on the surface of TAMS to be retained. This is favorable for the grafting of dopamine, which subsequently enhances the ability of the dopamine-functionalized TAMS to immobilize biomolecules. However, to the best of our knowledge, dopamine-functionalized tannic acid-templated mesoporous silica (Dop-TAMS) has never been used as an enzyme immobilization support.

**Figure 1:**
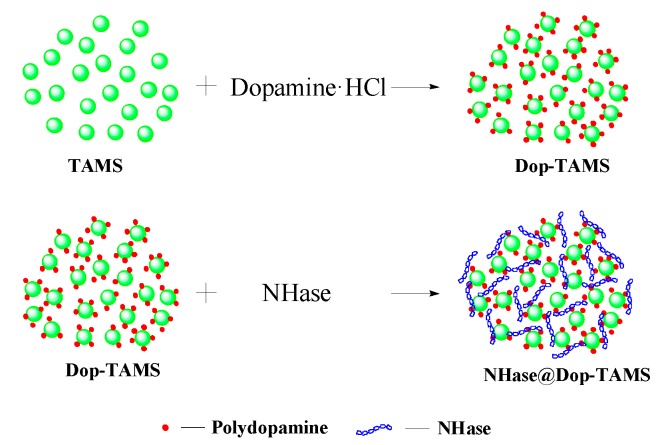
The reaction scheme for immobilization of NHase in dopamine-functionalized tannic acid-templated mesoporous silica (Dop-TAMS).

Nitrile hydratase (NHase; EC 4.2.1.84) is a kind of microbial enzyme, which catalyzes the hydration of nitriles to high value amides, with extensive applications in industrial production processes [28]. The use of free NHase in industrial applications is not conducive to improving efficiency due to low stability, poor adaptability to environment, and poor recovery rate [6]. Therefore, immobilization of NHase on a suitable support is very important. Thus, in this work, the Dop-TAMS was synthesized, and NHase was immobilized in Dop-TAMS by physisorphtion (named NHase@Dop-TAMS). The reaction scheme for immobilization of NHase in Dop-TAMS is shown in Figure 1. Additionally, the activity, operational stability, and reusability of NHase@Dop-TAMS were investigated detailedly. The results showed that the Dop-TAMS had great potential for the immobilization of NHase in practical applications.

## 2. Results and Discussion

### 2.1. Characterization of the Dop-TAMS and NHase@Dop-TAMS

**Figure 2:**
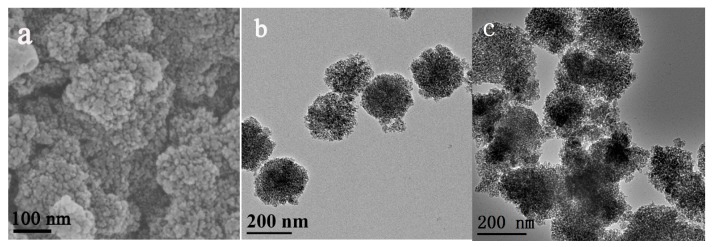
(**a**) SEM and (**b**) TEM images of Dop-TAMS and (**c**) TEM image of NHase@Dop-TAMS.

**Figure 3:**
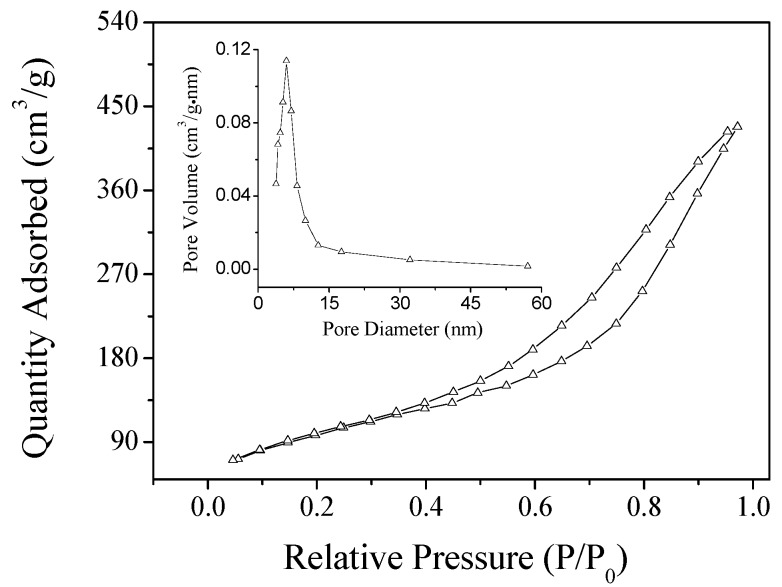
N_2_ adsorption/desorption isotherms and pore size distribution of the Dop-TAMS.

The SEM and TEM images of Dop-TAMS are shown in Figure 2. Spherical and monodisperse particles can be viewed from the typical SEM photograph of Dop-TAMS (Figure 2a) and the average diameter of the monodisperse particles was about 120–180 nm. The TEM image clearly exhibits that the Dop-TAMS had porous structure with irregular pore arrangement (Figure 2b), and after immobilization of NHase, the NHase@Dop-TAMS exhibited a small amount of aggregation (Figure 2c). Figure 3 shows the N_2_ adsorption/desorption isotherms and pore size distribution of the Dop-TAMS. According to the BET surface area measurements, the specific surface area of Dop-TAMS was 389 m^2^/g and the calculated BJH pore volume and pore size were 0.71 cm^3^/g and 6.1 nm, respectively. The large pore size and pore volume were beneficial for the enzyme entering the internal structure of the Dop-TAMS and ensuring the accommodation of NHase.

**Figure 4:**
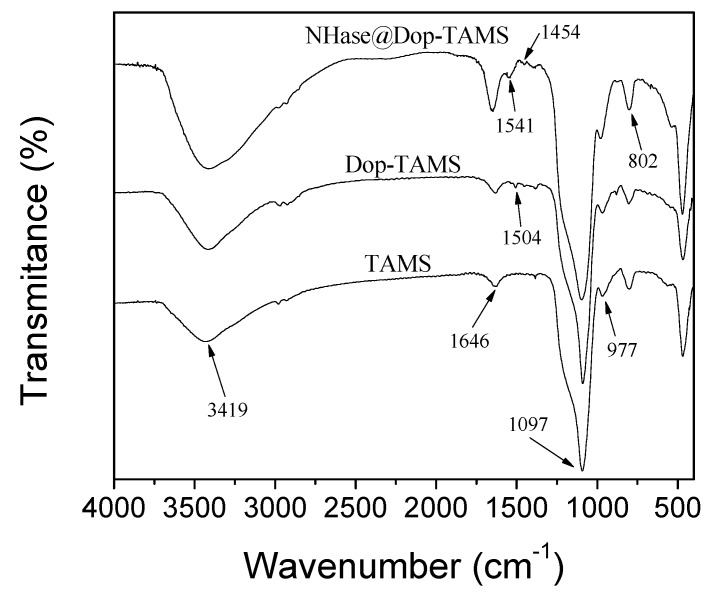
FT-IR spectra of the TAMS, Dop-TAMS, and NHase@Dop-TAMS.

Figure 4 shows the FT-IR spectra of TAMS, Dop-TAMS, and NHase@Dop-TAMS. The peaks at 1646 cm^−1^ and 3419 cm^−1^ were attributed to the stretching vibration of water molecules and hydroxyl, respectively [29]. The typical peaks at 1097 cm^−1^ and 802 cm^−1^ corresponded to the asymmetric stretching and symmetric stretching of Si-O-Si bonds, respectively [30]. The adsorption band at 977 cm^−1^ was attributed to the symmetric stretching of Si-OH groups [31]. The adsorption peak at 1504 cm^−1^ of Dop-TAMS was attributed to benzene ring stretching from dopamine [32,33], which demonstrated its successful graft. For the NHase@Dop-TAMS, the peaks at 1541 cm^−1^ and 1454 cm^−1^ were due to the deformation vibration of the amino groups and the bending vibration of the -C-H groups [3], which proved that the NHase was successfully immobilized in the Dop-TAMS.

The enzyme loading capacities and activities of NHase@TAMS and NHase@Dop-TAMS are shown in Table 1. According to the results, when the initial concentration of NHase in the phosphate buffer solution was 1.04 mg/mL and the dosage of TAMS was 10 mg/mL, the amount of NHase immobilized in NHase@Dop-TAMS was 63.1 mg/g and the activity recovery was 40.9%. In the control experiment, the enzyme loading amount of NHase@TAMS was 47.7 mg/g and the activity recovery was 35.2%. The improved NHase loading capacity and specific activity of NHase@Dop-TAMS could be attributed to the catechol and primary amines in dopamine molecules reacting with the NHase molecules to form strong conjugation of covalent bonds.

### 2.2. Thermal Stability of Free and Immobilized NHase

**Figure 5:**
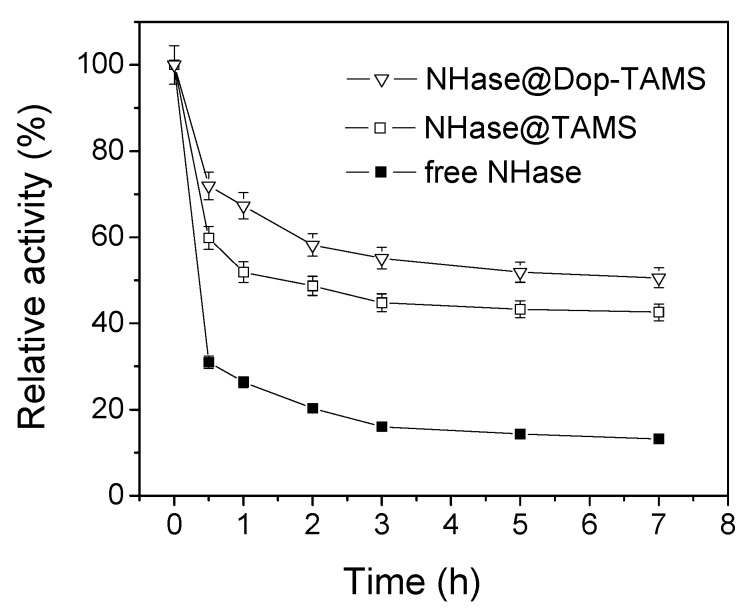
The thermal stability of free and immobilized NHase.

Figure 5 shows the thermal stabilities of free NHase and immobilized NHase inphosphate buffer solution (50 mM, pH 7.0) over different times at 50 °C. The rate of decrease in NHase@Dop-TAMS was lower than in free NHase and NHase@TAMS. After 7 h of incubation at 50 °C, the relative activity of free NHase and NHase@TAMSretained 13.2% and 42.6%, respectively, whereas the relative activity of NHase@Dop-TAMS maintained 50.7%. These results indicate that the NHase@Dop-TAMS had better thermal stability than the free NHase and NHase@TAMS, and this can be mainly attributed to the fact that Dop-TAMS can enhance the rigidity of NHase and protect it from unfolding, thus preventing denaturation of NHase molecules at high temperatures [34,35,36].

### 2.3. pH Stability of Free and Immobilized NHase

**Figure 6:**
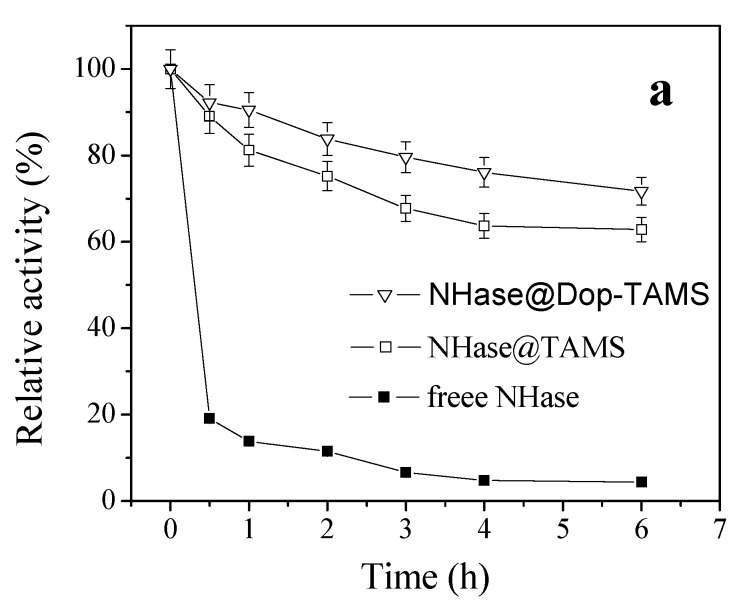

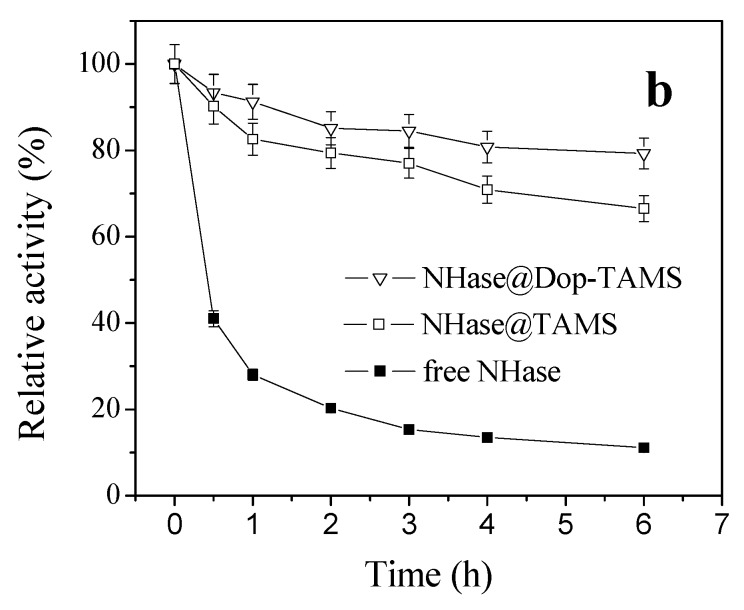
pH stability of free NHase and immobilized NHase at (**a**) pH 3.0; and (**b**) pH 9.0.

The pH stabilities of free NHase and immobilized NHase were mensurated by incubating them in phosphate buffer solutions at pH 3.0 and pH 9.0, respectively, and the results are shown in Figure 6. After 0.5 h of incubation at pH 3.0 or pH 9.0, the relative activity of the free NHase declined quite sharply; in constrast, the decreases in relative activity for the immobilized NHase varieties were considerably smaller. After 6 h of incubation at pH 3.0, the free NHase and NHase@TAMS retained 4.5% and 62.8% of their original activity, respectively, and the remaining activity of NHase@Dop-TAMS was 71.7%. After 6 h of incubation at pH 9.0, the relative activity of free NHase and NHase@TAMS retained 11.2% and 66.5%, respectively, and that of NHase@Dop-TAMS retained 79.3%. Therefore, under both acidic or alkaline conditions, NHase@Dop-TAMS was more stable than free NHase or NHase@TAMS. This might be attributed to the strong conjugation of covalent bonds between NHase and dopamine-functionalized TAMS, which can restrain the conformational change of the NHasemolecules caused by H^+^ or OH^−^ [6,37,38].

### 2.4. Stability of Free and Immobilized NHase in Shaking Conditions

**Figure 7:**
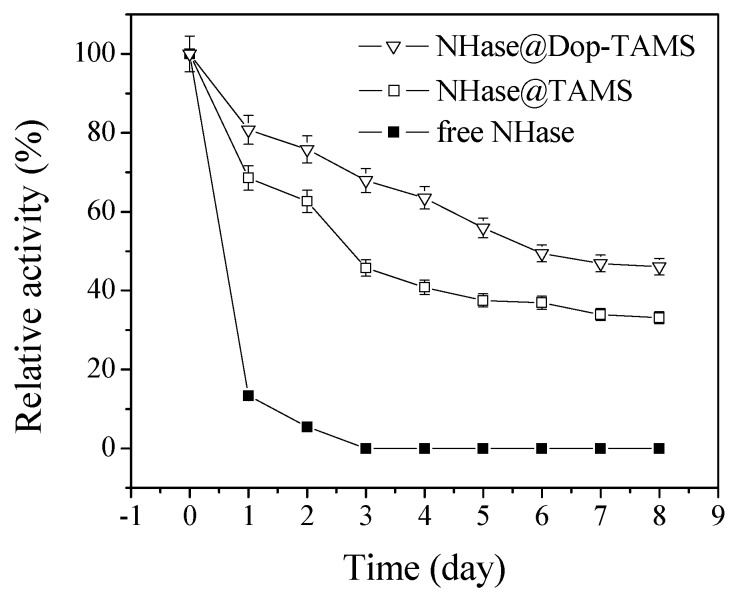
The stability of free and immobilized NHasein shaking conditions.

Figure 7 shows the stability of free NHase andimmobilized NHase in shaking conditions (200 rpm). The relative activity of free NHase varied tremendously and decreased to zero after shaking for 3 d and the adsorption of NHase in TAMS or Dop-TAMS improved its stability. Under the same conditions, the relative activity of NHase@TAMS and NHase@Dop-TAMS could maintain 33.4% and 46.1%, respectively, after 8 d. The decrease of activity in free and immobilized NHase was because vigorous shaking led to the autolysis and denaturation of the enzyme molecules [6,39]. Compared to NHase@TAMS, the improved storage stability of NHase@Dop-TAMS might be attributed to strong conjugation of covalent bonds between the NHase and dopamine-functionalized TAMS, which would not only prevent leaching of NHase from NHase@Dop-TAMS, but also increase conformational stability of the NHase molecules [39,40,41].

### 2.5. Storage Stability of Free and Immobilized NHase

**Figure 8:**
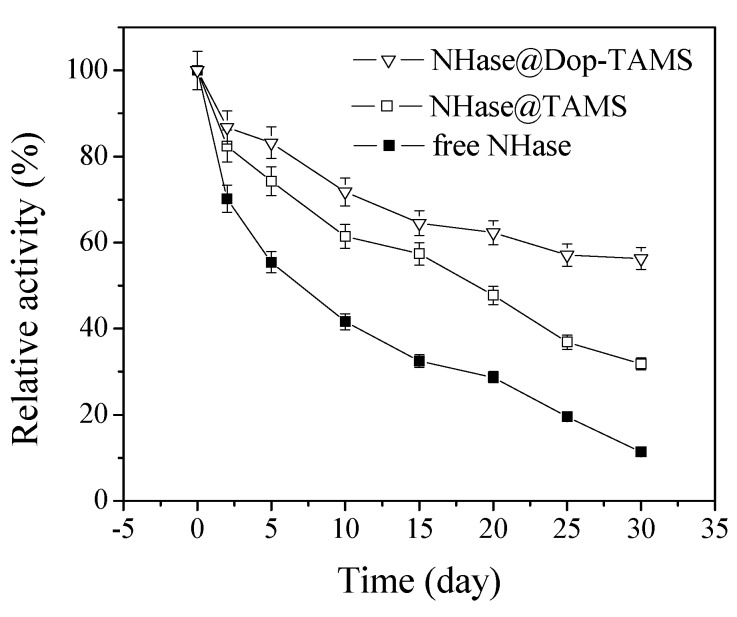
The storage stability of free and immobilized NHase.

The storage stability of enzymes is also a crucial factor in practical applications [42]. Figure 8 shows the storage stability of the free NHase and immobilized NHase. It was observed that the activity of the free NHase decreased faster than that of the immobilized NHase. After being stored for 30 d, the free NHase and NHase@TAMS maintained 11.4% and 31.8% of their initial activities, respectively, whereas the residual activity of the NHase@Dop-TAMS maintained 56.3%. Thus, the investigation proved that the immobilization of NHase in Dop-TAMS could prolong its storage life. The improved storage stability of NHase@Dop-TAMS might be related to protecting the active sites of the enzyme from deactivating more effectively, compared with free NHase under the same conditions [36,38].

### 2.6. Kinetics of Free and Immobilized NHase

The kinetic parameters of freeNHase and immobilized NHase are shown in Table 2. The K_m_ values of the free NHase, NHase@TAMS and NHase@Dop-TAMS were 1.71 mM, 2.48 mM, and 2.35 mM, respectively. Distinctly, the immobilized NHase exhibited a higher K_m_ value than the free NHase, demonstrating that the substrate’s affinity capacity for the NHase@TAMS or NHase@Dop-TAMS was lower than for the free NHase. This might be ascribed to the conformational change of the NHase after its immobilization, which could lower the substrate’s accessibility toward the active sites of the immobilized NHase [43,44]. The K_m_ value of the NHase@Dop-TAMS was lower than that of the NHase@TAMS, suggesting that the NHase@Dop-TAMS could enhance the NHase’s affinity capacity for the substrate. The V_max._ values for the NHase@TAMS and NHase@Dop-TAMS were lower than for the free NHase, indicating that while the substrate diffused and accessed the enzyme molecules, the mass transfer limitation of the immobilized NHase was larger than for the free NHase [42]. Moreover, the V_max._ value of NHase@Dop-TAMS was higher than for NHase@TAMS, demonstrating that the polydopamine on the surface of Dop-TAMS enhanced the catalytic ability of NHase@Dop-TAMS [38].

### 2.7. Reusability of Immobilised NHase

**Figure 9:**
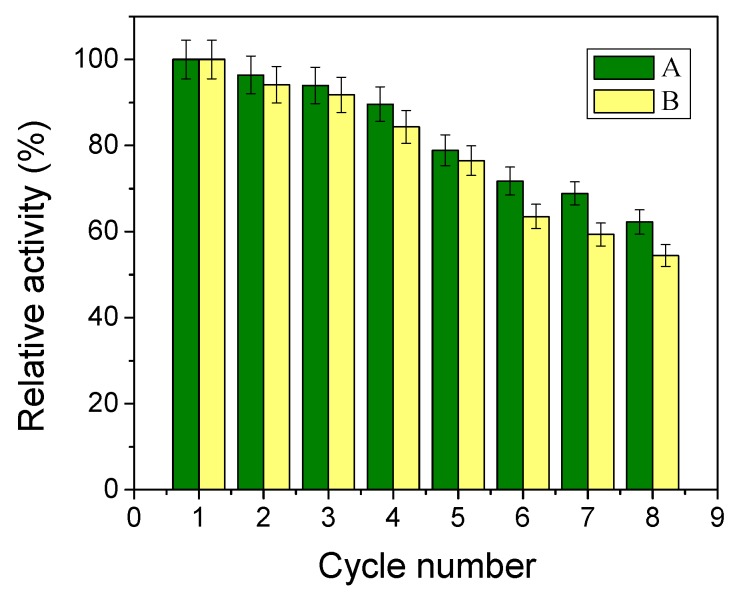
The reusability of (**A**) NHase@Dop-TAMS; and (**B**) NHase@TAMS.

Studying the reusability of the immobilized NHase is very meaningful for large-scale industrial applications. Figure 9 shows the results for reusability of NHase@TAMS and NHase@Dop-TAMS. After recycling for 8 batches, the relative activity ofNHase@TAMS and NHase@Dop-TAMS was found to be 52.4% and 62.2%, respectively. The activity loss for the enzyme can be primarily attributed to leakage of NHase from the carrier and denaturation of the NHase molecules [19,23]. Compared with the NHase@TAMS, the activity of NHase@Dop-TAMS decreased slowly, and the improved operational stability of NHase@Dop-TAMS can be ascribed to the strong conjugation of covalent bonds between the NHase molecules and the polydopamine on the surface of Dop-TAMS, which could have prevented the leakage of NHase from the NHase@Dop-TAMS [36]. The favorable reusability of NHase@Dop-TAMScould reduce the cost of biocatalysts and improve economic benefits in practical applications.

## 3. Materials and Methods

### 3.1. Materials

Dopamine hydrochloride and tannic acid were purchased from Sigma-Aldrich (Shanghai, China). Tetraethoxysilane (TEOS) and ammonium hydroxide were purchased from Meryer (Shanghai, China). NHase was purchased from Hangzhou Biosci Biotech Co. (Hangzhou, China). Ethanol was purchased from Sinopharm Chemical Reagent Co., Ltd. (Shanghai, China). Acrylonitrile was purchased from Alfa Aesar (Shanghai, China). All other chemical reagents were of AR grade and used as received.

### 3.2. Preparation of Dopamine-Functionalized TAMS (Dop-TAMS)

TAMS was synthesized using tannic acid as the template, according to the reported method [18]. Dopamine-functionalized TAMS was synthesized using the post-grafting method. Typically, 1.0 g of TAMS was added into 200 mL of 1 g/L dopamine solution, which was freshly prepared in phosphate buffer (pH 8.5), and the suspension was stirred for 3 h. Then the slurry was centrifuged and washed with phosphate buffer (pH 8.0) three times. The material prepared in this manner was denoted as Dop-TAMS.

### 3.3. Immobilization of NHase in Dop-TAMS

To immobilize NHase in Dop-TAMS, 50 mg of Dop-TAMS was mixed with 5 mL of NHase phosphate buffer solution (1.04 mg/mL, 0.1 M PBS, pH 7.0). The resulting solutionwas kept under constant stirring (200 rpm) at ambient temperature for several hours. After this step, the Dop-TAMS loading NHase was separated from the solution by centrifugation and washed with PBS (0.1 M, pH 7.0) several times. The resulting product was denoted as NHase@Dop-TAMS, which was stored in PBS at 4 °C until use. The quantity of NHase in the solution was measured by Bradford assay and the loading amounts of NHase in the matrix were calculated [23]. NHase was also immobilized in TAMS by physical adsorption (named NHase@TAMS), in order to make comparisons with the NHase@Dop-TAMS.

### 3.4. Enzymatic Activity Assay

Enzymatic activities offree NHase or immobilized NHase were assayed according to the reported procedure [6,7,45]. Specifically, an appropriate amount of native NHase or immobilized NHase was added to 125 mM of acrylonitrile solution prepared in phosphate buffer (50 mM, pH 7.0) and the suspension was stirred for 5 min at 30 °C. Finally, 0.2 mL of 0.6 M HCl was added to the mixture to stop the reaction. High-performance liquid chromatography (HPLC, 600E-2487, Waters, Milford, MA, United States) was utilized to measure the formed acrylamide concentration. One unit of NHase activity was defined as the amount of NHase which released 1 µM of acrylamide per minute under the above conditions.

### 3.5. Thermal and pH Stability

For thermal stability studies, free NHase or immobilized NHase was incubated in PBS (50 mM, pH 7.0) at 50 °C for several hours and the residual activities were measured periodically by the method described in Section 2.4. The relative activity was calculated from the ratio of the residual activity to the initial activity.

pH stabilities were assayed by incubating the free NHase or immobilized NHase in pH 3.0 and pH 9.0 PBS at 25 °C for 6 h and, after each incubation period, the residual activities were measured. The pH of the PBS was adjusted by 0.1 M NaOH or 0.1 M HCl and the relative activities werecalculated as described above.

### 3.6. Stability of Free and Immobilized NHase in Shaking and Storage Conditions

The stabilities in shaking conditions were determined by shaking the free NHase or immobilized NHase (200 rpm) in pH 7.0 phosphate buffer solutions at room temperature for several days and the residual activities were measured periodically. The relative activities were therefore calculated.

In order to determine storage stability, the free NHase or immobilized NHase were immersed in pH 7.0 phosphate buffer solutions at 4 °C for several days. The residual activities were measured periodically and the relative activities were calculated.

### 3.7. Determination of Kinetic Parameters

The kinetic parameters of freeNHase and immobilized NHase were calculated by using the Michaelis–Menten equation (1/V versus 1/[S]). The reactions were carried out using acrylonitrile as substrate in the initial concentration ranges of 2 to 50 mM. At the end of the reaction, the acrylamide yields were measured by the HPLC method.

### 3.8. Reusability

The reusability of NHase@TAMS and NHase@Dop-TAMS were determined according to the enzyme activity assay method described in Section 2.4. At the end of each batch reaction, the immobilized NHase was centrifuged, washed with PBS (50 mM, pH 7.0), and then reused in the next reaction.

### 3.9. Characterizations

The scanning electron microscopy (SEM) images of Dop-TAMS were gained by a Hitachi S-4800 SEM (Tokyo, Japan). The transmission electron microscopy (TEM) images were gained by a JEOL JEM-2100F TEM (Tokyo, Japan) at 200 kV. The Brunauer-Emmett-Teller (BET) surface area was calculated according to the adsorption data, which was measured by a BEL BET BELSORP-max, and the pore size distributions were obtained according to the Barrett-Joyner-Halenda (BJH) method. The Fourier transform infrared (FT-IR) spectra of TAMS, Dop-TAMS and immobilized NHase were recorded using a Bruker VECTOR22 Fourier transform infrared spectrometer (Karlsruhe, Germany) by KBr pellet.

## 4. Conclusions

In summary, tannic acid-templated mesoporous silica (TAMS) was synthesized using a simple, cost-effective, and environmentally-friendly nonsurfactant template method and dopamine-functionalized TAMS (Dop-TAMS) was developed as a carrier to immobilize NHase (NHase@Dop-TAMS). The study results suggest that the thermal stability, storage stability, shaking stability, and pH stability of the NHase@Dop-TAMS were significantly enhanced. Furthermore, the NHase@Dop-TAMS showed good reusability. Based on these advantages, the efficient immobilization of NHase in Dop-TAMS has great potential in practical applications.

## Figures and Tables

**Table 1:** The enzyme loading capacities and activities of NHase@TAMS and NHase@Dop-TAMS.

Samples	NHase Loading Capacity (mg/g)	Specific Activity (U/mg)	Activity Recovery (%)
NHase@Dop-TAMS	63.1	2.14	40.9
NHase@TAMS	47.7	1.85	35.2

**Table 2:** Kinetic parameters of free and immobilised NHase.

Samples	Free NHase	NHase@TAMS	NHase@Dop-TAMS
*K_m_* (mM)	1.71	2.48	2.35
*V_max._* (mM/min)	4.46	3.59	3.96
*V_max._*/*K_m_* (s^−1^)	2.61	1.45	1.69

## Supplementary Materials

Supplementary File 1
