# Supplementary material for: Biomimetic-Functionalized, Tannic Acid-Templated Mesoporous Silica as a New Support for Immobilization of NHase
Source: Molecules. 2017 Sep 25;22(10):1597. doi: 10.3390/molecules22101597 (PMC6151425; doi:10.3390/molecules22101597)
Supplement: Supplementary File 1 [file molecules-22-01597-s001.pdf]

We certify that the following article

Biomimetic functionalized tannic-acid-templated mesoporous silica as a new support for immobilization of NHase

Junkai Gao, Zijun Zhang, Yanjun Jiang, Yan Chen *, Shufeng Gao

has undergone English language editing by MDPI. The text has been checked for correct use of grammar and common technical terms, and edited to a level suitable for reporting research in a scholarly journal. MDPI uses experienced, native English speaking editors. Full details of the editing service can be found at ► www.mdpi.com/authors/english.

Multidisciplinary Digital Publishing Institute

Basel, September 2017

Martyn Rittman, Ph.D.
English Editing Manager
